# Comparison of virus isolation using the Vero E6 cell line with real-time RT-PCR assay for the detection of human metapneumovirus

**DOI:** 10.1186/1471-2334-10-170

**Published:** 2010-06-14

**Authors:** Yoko Matsuzaki, Katsumi Mizuta, Emi Takashita, Michiko Okamoto, Tsutomu Itagaki, Fumio Katsushima, Yuriko Katsushima, Yukio Nagai, Hidekazu Nishimura

**Affiliations:** 1 Course of Clinical Nursing, Yamagata University Faculty of Medicine, Iida-nishi, 2-2-2, Yamagata, 990-9585, Japan; 2 Department of Microbiology, Yamagata Prefectural Institute of Public Health, Tokamachi 1-6-6, Yamagata, 990-0031, Japan; 3 Influenza Virus Research Center, National Institute of Infectious Diseases, Gakuen 4-7-1, Musashimurayama, Tokyo, 208-0011, Japan; 4 Virus Research Center, Clinical Research Division, Sendai Medical Center, Miyagino-ku Miyagino 2-8-8, Sendai, 983-8520, Japan; 5 Yamanobe Pediatric Clinic, Yamanobe 2908-14, Yamagata, 990-0301, Japan; 6 Katsushima Pediatric Clinic, Minamidate 4-4-12, Yamagata, 990-2461, Japan; 7 Nagai Children's Clinic, Miyagino-ku Miyagino 1-25-10, Sendai, 983-0045, Japan; 8 Department of Infectious Diseases, Yamagata University Faculty of Medicine, Iida-nishi 2-2-2, Yamagata, 990-9585, Japan; 9 Department of Virology Tohoku University Graduate School of Medicine, Seiryo-machi 2-1, Aoba-ku, Sendai, 980-8575, Japan

## Abstract

**Background:**

The use of cell culture for the diagnosis of human metapneumovirus (hMPV) infection is uncommon at present and molecular method such as reverse-transcription PCR (RT-PCR) has been widely and most commonly used as the preferred test. We aimed to compare the results of virus isolation using Vero E6 cells with real-time RT-PCR for the detection of hMPV, since such a comparison data is not available.

**Methods:**

Between December 2007 and July 2008, we obtained 224 nasopharyngeal swab specimens from patients with acute respiratory infection and tested by the two methods.

**Results:**

Forty-three (19.2%) were found positive by cell culture and 62 (27.7%) by real-time RT-PCR. Cell cultures were positive for 42 of 62 specimens found positive by real-time RT-PCR (67.7% sensitivity) and for 1 of 162 specimens found negative by real-time RT-PCR (99.4% specificity), respectively. The sensitivity of the cell culture was 76.2-87.5% (mean 81.8%) when specimens were collected within 3 days after the onset of symptoms, and the sensitivity decreased to 50% or less thereafter. Among specimens collected within 3 days after symptom onset, all of the real-time RT-PCR positive specimens having a viral load of more than 1.25×10^5 ^copies/ml were found positive by cell culture.

**Conclusions:**

Cell culture using Vero E6 cell line has 81.8% sensitivity compared with the real-time RT-PCR method, when specimens are collected within 3 days after the onset of symptoms. Thus, this method is a useful method for epidemiological and virological research even in facilities with minimal laboratory resources.

## Background

Human metapneumovirus (hMPV) was first described in 2001 following its isolation from nasopharyngeal specimens from infants and children with acute respiratory infection (ARI) in The Netherlands and it has been categorized as a member of the genus *Metapneumovirus *of the subfamily *Pneumovirinae *of the family *Paramyxoviridae *[1-3]. It has been recognized as an important agent responsible for respiratory tract disease worldwide, especially in the pediatric and elderly populations [2-5], with serological studies having revealed that hMPV seropositivity is almost universal by the age of 5 years [3,6].

For laboratory diagnosis, the most definitive test is virus isolation by cell culture [3]. Although hMPV isolation has commonly been performed using the LLC-MK2 or Vero cell lines, the growth of hMPV is slow and often requires several blind passages before any cytopathic effect (CPE) is apparent, particularly following primary isolation [1,3-5,7,8]. Thus, it is believed that virus isolation is difficult, being possible in only one-third to one-half of cases in which nasopharyngeal samples are found to positive for hMPV by reverse-transcription PCR (RT-PCR) [2,3,5,9]. The use of cell culture for the diagnosis of hMPV infection is uncommon at present and RT-PCR has been widely and most commonly used as the preferred test due to its sensitivity [2,3]. In our previous study, we demonstrated that the infection efficiency of hMPV in Vero E6 cells was more than 5 to 20 times better than that in LLC-MK2 cells and, in fact, we succeeded in isolating nearly 100 strains between 2004 and 2006 using this cell line [6,10,11]. However, there is no data on the effectiveness of virus isolation in comparison with real-time RT-PCR for hMPV detection from clinical specimens, since virus isolation is not common. In this paper, we show the results of virus isolation from 224 clinical specimens using Vero E6 cells and the real-time RT-PCR method.

## Methods

A total of 224 nasopharyngeal swab specimens were collected from patients who had been clinically diagnosed with ARI with fever and/or cough and/or rhinorrhea at 3 pediatric clinics in Yamagata and Sendai between December 2007 and July 2008, as described previously for the evaluation of a newly developed rapid antigen detection kit using immunochromatography compared with real-time RT-PCR [12]. The specimens were collected and used for the previous and present studies, after getting the informed consent from the patients or their guardians. These studies were approved by the Institutional Review Board of Sendai Medical Center, National Hospital Organization Japan, on Feb. 29, 2008 (Reference No CHI-19-33). Of these specimens, 166 were from children aged under 5 years, 43 were from children aged between 5 and 9 years, 7 were from children aged between 10 and 15 years and 8 were from patients > 15 years. Each specimen was placed immediately in a tube containing 3 ml of transport medium [11,12] and transported at 4°C to the Department of Microbiology, Yamagata Prefectural Institute of Public Health for virus isolation and real-time RT-PCR. Prior to specimen inoculation for virus isolation, 200 μl of the specimen was transferred to a 1.5 ml microtube and stocked at -80°C until application to real-time RT-PCR assay. Virus isolation was carried out by the 96-well modified microplate method including Vero E6 and other five cell lines [10,11]. We observed the plates two or three times per week for CPEs for 4 weeks without passage or medium change [10]. When a hMPV-like CPE was observed, second passage, identification and genotyping was carried out as described previously [10]. The method and results of real-time RT-PCR assay with a TaqMan probe using an ABI Prism 7500 Fast real-time PCR system (Applied Biosystems) were described previously [12]. Primers and a probe targeting the hMPV N gene were designed based on the reports of Maertzdorf et al. [13] and Bonroy et al. [14]. Statistical analysis was performed by Fisher's exact probability test.

## Results

As a result, 62 specimens (27.7%) were found positive by real-time RT-PCR, and 43 (19.2%) by cell culture as shown in Table 1. One of the 43 cell culture-positive specimen was found negative by real-time RT-PCR. This may be a false-positive result obtained by cell culture or rather a false-negative by real-time RT-PCR. On the other hand, 20 of the 62 real-time RT-PCR-positive specimens were found negative by cell culture. Therefore, the sensitivity and specificity of cell culture for the detection of hMPV were 67.7% (42/62) and 99.4% (161/162), respectively, compared with real-time RT-PCR. The agreement between the results of cell culture and real-time RT-PCR was 90.6% (203/224).

**Table 1:** Comparison of virus isolation with real-time RT-PCR in hMPV detection

Days of specimen collection after onset of illness	No. of specimens	No. of specimens with the following results^a^:				Sensitivity	Specificity
		P+,C+	P+,C-	P-,C+	P-,C-	(%)	(%)
1	48	13	2	0	33	86.7	100
2	77	16	5	0	56	76.2	100
3	38	7	1	0	30	87.5	100
4	25	3	3	1	18	50	94.7
> 4	36	3	9	0	24	25	100
							
1-7	224	42	20	1	161	67.7	99.4

^a^Abbreviations; P, real-time RT-PCR; C, cell culture

**Figure 1:**
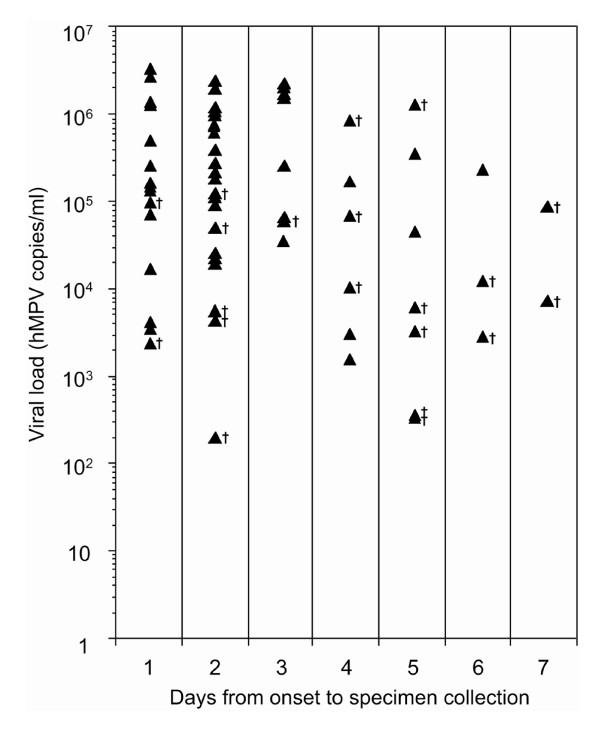
**Viral loads of real-time RT-PCR-positive specimens and the number of days from onset of fever to specimen collection**. Solid triangles indicate the 62 specimens that tested positive for hMPV by real-time RT-PCR assay. Specimens testing negative by cell culture are marked with daggers.

Viral loads of the 62 specimens tested positive by real-time RT-PCR were distributed between 1.99 × 10^2 ^and 3.85 ×10^6^. The results of the real-time RT-PCR are shown in Figure 1. All of the real-time RT-PCR-positive specimens having more than 1.25×10^5 ^hMPV copies/ml were also found positive by cell culture, though two specimens with 8.79×10^5 ^and 1.36×10^6 ^copies/ml that were collected on Day 4 and 5 after the onset of symptoms, respectively, were negative. The sensitivity of virus isolation for specimens obtained at 1-3 days after the onset of symptoms was 76.2-87.5% (mean 81.8%), whereas it was less than 25-50% (mean 33.3%) for 4-7 days (Table 1). The sensitivity of virus isolation for specimens obtained within 3 days after the onset of symptoms was statistically higher than that for specimens obtained more than 3 days after the onset of symptoms (*P *= .0006).

## Discussion

Since the date of specimen collection is important, we should distinguish the data obtained at 1-3 days after the onset of symptoms from thereafter, when we compare virus isolation with real-time RT-PCR method. The sensitivity of virus isolation for specimens during this period was 76.2-87.5% (mean 81.8%). However, the sensitivity decreased for specimens collected on Day 4 and thereafter. This indicates that the excretion of hMPV infectious particles may decrease after 4 days from the onset of symptoms, which is in agreement with previous data for respiratory syncytial virus that indicates the predictive value of virus isolation is highest when respiratory specimens are collected 1-3 days after the onset of symptoms [15]. Therefore, specimens for cell culture should be collected within three days after the onset of symptoms. The merit of real-time RT-PCR over cell culture rests in that it is able to detect hMPV genome with or without infectivity during this period. Since the two detection methods described here differ significantly in cost, turn-around-time and technical difficulty, we should use each method depending on the purpose.

## Conclusions

Cell culture using Vero E6 cell line has approximately 80% sensitivity compared with the real-time RT-PCR method when specimens are collected within 3 days after the onset of symptoms. Along with its consistency in not producing false-positive results, cell culture using the Vero E6 cell line to be regarded as a useful method for epidemiological and virological research, through not for rapidly clinical diagnosis. Furthermore, this cell culture method might be applicable in facilities with minimal laboratory resources without expensive real-time RT-PCR system.

## Abbreviations

HMPV: human metapneumovirus; ARI: acute respiratory infection; CPE: cytopathic effect; RT-PCR: reverse-transcription PCR

## Competing interests

The authors declare that they have no competing interests.

## Authors' contributions

All authors have made substantial contributions to design and acquisition of data. YM, KM and HN also contributed for analysis of data, drafting and revising the manuscript. All authors read and approved the final manuscript.

## Pre-publication history

The pre-publication history for this paper can be accessed here:

http://www.biomedcentral.com/1471-2334/10/170/prepub
